# Effect of 12 weeks of yoga training on the somatization, psychological symptoms, and stress-related biomarkers of healthy women

**DOI:** 10.1186/1751-0759-8-1

**Published:** 2014-01-03

**Authors:** Kazufumi Yoshihara, Tetsuya Hiramoto, Takakazu Oka, Chiharu Kubo, Nobuyuki Sudo

**Affiliations:** 1 Department of Psychosomatic Medicine, Graduate School of Medical Sciences, Kyushu University, 3-1-1 Maidashi, Higashiku 812-8582, Fukuoka, Japan; 2 Division of Cerebral Integration, Department of Cerebral Research, National Institute for Physiological Sciences, 38 Myodaiji-Nishigonaka, Okazaki 444-8585, Aichi, Japan

**Keywords:** Yoga, Somatization, Psychological symptom, Stress, Biomarker, Anxiety, Depression, Anger, Hostility, Fatigue

## Abstract

**Background:**

Previous studies have shown that the practice of yoga reduces perceived stress and negative feelings and that it improves psychological symptoms. Our previous study also suggested that long-term yoga training improves stress-related psychological symptoms such as anxiety and anger. However, little is known about the beneficial effects of yoga practice on somatization, the most common stress-related physical symptoms, and stress-related biomarkers. We performed a prospective, single arm study to examine the beneficial effects of 12 weeks of yoga training on somatization, psychological symptoms, and stress-related biomarkers.

**Methods:**

We recruited healthy women who had no experience with yoga. The data of 24 participants who were followed during 12 weeks of yoga training were analyzed. Somatization and psychological symptoms were assessed before and after 12 weeks of yoga training using the Profile of Mood State (POMS) and the Symptom Checklist-90-Revised (SCL-90-R) questionnaires. Urinary 8-hydroxydeoxyguanosine (8-OHdG), biopyrrin, and cortisol levels were measured as stress-related biomarkers. The Wilcoxon signed-rank test was used to compare the stress-related biomarkers and the scores of questionnaires before and after 12 weeks of yoga training.

**Results:**

After 12 weeks of yoga training, all negative subscale scores (tension-anxiety, depression, anger-hostility, fatigue, and confusion) from the POMS and somatization, anxiety, depression, and hostility from the SCL-90-R were significantly decreased compared with those before starting yoga training. Contrary to our expectation, the urinary 8-OHdG concentration after 12 weeks of yoga training showed a significant increase compared with that before starting yoga training. No significant changes were observed in the levels of urinary biopyrrin and cortisol after the 12 weeks of yoga training.

**Conclusions:**

Yoga training has the potential to reduce the somatization score and the scores related to mental health indicators, such as anxiety, depression, anger, and fatigue. The present findings suggest that yoga can improve somatization and mental health status and has implications for the prevention of psychosomatic symptoms in healthy women.

**Trial registration:**

University Hospital Medical Information Network (UMIN CTR) UMIN000007868.

## Background

Yoga is an ancient technique used for promoting physical and mental health through postures, the regulation of breathing, and meditation. Studies have shown that the practice of yoga reduces perceived stress and negative feelings and that it improves psychological symptoms by lowering the levels of anxiety and anger [1-5]. We also showed that long-term yoga training improves stress-related psychological symptoms, such as anxiety and anger [6].

In addition to these stress-related psychological symptoms, somatization, the most common stress-related physical symptom, is frequently seen in clinical care settings. Somatization is defined as “a tendency to experience and communicate somatic distress in response to psychosocial stress and to seek medical help for it” [7]. Clinically significant somatization leads to excessive health care use. For example, it costs the US health care system an estimated over $100 billion annually [8]. These medically unexplained physical symptoms include headache, dizziness, chest pain, lower back pain, nausea, muscle soreness, breathing problems, hot or cold spells, numbness or tingling in parts of the body, lumps in the throat, a weak feeling in parts of the body, and a heavy feeling in the arms or legs. There are numerous treatments for somatization, and they have varying degrees of effectiveness.

A few studies have demonstrated that somatization symptoms after mindfulness training, which includes meditation, were significantly decreased in comparison with before mindfulness training. Rosenzweig et al. indicated that greater home meditation practice was associated with improvement of somatization symptoms among participants with chronic pain conditions [9]. Franco et al. reported reduced teacher somatization scores on the Symptom Checklist-90-Revised (SCL-90-R) through a mindfulness training program [10]. With regard to yoga, only one study in the English literature has shown the potential effects of yoga practice on somatization symptoms [11]. Although Telles et al. reported that the somatization score was reduced after one week of yoga practice (Before yoga: 10.93 ± 6.05, After yoga: 7.03 ± 5.90) [11], the baseline somatization score on the SCL-90-R in a yoga group of healthy volunteers was higher compared with that of a control group (Yoga group: 10.93 ± 6.05, Control group: 5.69 ± 5.66). There is little evidence about the effects on somatization symptoms of yoga that includes a combination of classical postures, breathing exercises, and meditation.

Because there are no clear biomarkers capable of objectively measuring psychosocial distress, many researchers have examined self-rating symptom scores using questionnaires and various stress-related biomarkers, such as cortisol and catecholamine. Recently, stress-related urinary biomarkers, such as 8-hydroxydeoxyguanosine (8-OHdG) and biopyrrin, have been used to assess psychological distress because blood sampling itself is invasive and has been associated with psychological stress [12-14]. The urinary 8-OHdG level is a putative biomarker of total systemic oxidative stress [15], and psychological distress is associated with oxidative damage [12]. Biopyrrin, an oxidative metabolite of bilirubin, is a stress-related urinary biomarker [13,14,16]. In our previous study, we indicated that the urinary 8-OHdG concentration tended to be lower in a long-term yoga group than in a control group [6]. However, there have been no previous longitudinal studies showing that the practice of yoga simultaneously influences psychosomatic symptoms, urinary 8-OHdG, and biopyrrin levels.

In this study, we performed a prospective, single arm study to examine the beneficial effects for healthy individuals of a 12-week yoga program on indicators of somatization and psychological symptoms using the Profile of Mood States (POMS) and SCL-90-R questionnaires, and on stress-related biomarkers, such as urinary 8-OHdG, biopyrrin, and cortisol before initiation of a randomized control trial (RCT). We did not include patients with somatization symptoms because this is a pilot study. We tested the hypothesis that the indicators of somatization and psychological symptoms would be improved and that the levels of stress-related urinary biomarkers after 12 weeks of yoga training would be decreased compared with those before the start of yoga training.

In this paper, we used the data from a previous study of 38 healthy women with more than two years of experience with yoga (long-term yoga group) and 37 age-matched, healthy women who had no experience with yoga (control group) who were used to obtain reference data [6]. We hypothesized that the data for the psychological symptoms and the stress-related biomarkers of the yoga-training group before yoga training would be almost the same as the data for the control group. In addition, we assumed that there is a dose–response effect of yoga for several weeks and a longer threshold effect. Therefore, we hypothesized that the data on the psychological symptoms and the stress-related biomarkers of the yoga-training group after 12 weeks of yoga training would be almost the same as the data of the long-term yoga group.

## Methods

### Participants and data collection

A national survey of yoga practitioners reported that the percentage of females attending yoga training was 84.2% in the USA [17]. Because yoga is also far more popular for women than men in Japan, it seemed that it would be difficult to recruit men for a yoga study. Therefore, we recruited 39 healthy, adult women who had no experience with yoga. The participants were recruited by posters, flyers, and the Internet from ten yoga-training centers and from halls where yoga lessons were only a part of the lesson program. The recruitment sites were in Fukuoka, Kumamoto, and Kagoshima Prefectures of Japan. The following exclusion criteria were applied: (i) age < 20 years and > 50 years; (ii) taking medication including supplements in the month prior to the experiment; (iii) having an illness; and (iv) having a past history of significant physical or mental illness. All participants received detailed information on the purpose of the study and provided written informed consent. The participants who agreed were then handed questionnaires, a paper cup, a tube with a screw cap, and a self-addressed return parcel before and after the 12 weeks of yoga training. Each participant collected urine samples and, at the same time, answered the POMS and SCL-90-R questionnaires at home. The urine, frozen at home by the participant, and the questionnaires were sent as soon as possible via a parcel delivery service, which used a freezer van to keep the samples frozen at -18°C (-0.4°F). Participants who completed the yoga- training program received 2,000 yen (about $20). The study was approved by the Institutional Review Board of Kyushu University.

### Yoga intervention

Yoga classes were conducted one day a week for about one hour each session for 12 weeks. All instructors of the yoga classes are certificated. The participants were requested to attend at least 10 of the 12 weekly yoga sessions and to practice on their own at home at least twice a week for over 30 minutes during this 12-week period. If they could not attend 10 of the 12 classes or the requested home practice, they were dropped from the study. The self-reported home practice activity time was confirmed at a classroom session. Some yoga classes were closed classes, for only the participants of our study, and others were not. Of the various types of yoga, we chose cyclic meditation yoga as our yoga intervention because of the availability of scientific studies on this form of yoga (reviewed in [18]). At the beginning of the cyclic meditation yoga training, the instructors emphasized practicing slowly with awareness and relaxation. The cyclic meditation yoga consisted of the following practices;

1. Isometric contraction of the muscles of the body ending with rest in the supine position (Shavasana).

2. Standing at ease (Tadasana) and balancing the weight on both feet (Centering).

3. From the standing position (Tadasna), bending to the right and left (Ardhakati Cakrasana).

4. Forward bending (Pada Hastasana).

5. Backward bending (Ardha Chakrasana).

6. Supine posture for rest (Shavasana).

7. Bending the knees, holding them together while sitting down and adjusting the hips between the heels. While inhaling raise both arms above the head. While exhaling keep the back straight and bend the upper body and arms forward until the arms and forehead touch the floor, without raising the buttocks (Shashankasana).

8. From a kneeling position, coming up onto both knees and placing them hip width apart. Placing the palms of the hands on the sacrum with the fingers pointed down. Inhaling and pressing the knees down while extending the crown of the head up to lengthen the spine. Exhaling and pressing the hips forward, squeezing the buttocks and thighs, and supporting the body weight with the arms while bending backwards (Ardha Ushtrasana).

9. Slowly coming down to a supine posture for rest (Shavasana) with instructions to relax the body in sequence.

Although the participants sometimes practiced breathing exercises (Bhramari Pranayama) or meditation (Om meditation), they mainly practiced cyclic meditation yoga.

### Urine sampling

Urine samples were collected from all participants for the quantification of biopyrrin, 8-OHdG, cortisol, and creatinine. The participants were asked to avoid vigorous exercise and heavy psychological stress for 24 hours prior to urine collection. Urine was collected between 6:00 and 9:00 am, and 2 ml of urine from each participant was stored at -80°C (-112°F) until analysis. The urinary biopyrrin, cortisol, and 8-OHdG concentrations were measured by enzyme-linked immunosorbent assay kits (Shino-Test, Tokyo, Japan, Oxford Biomedical Research, Inc. MI, and Nikken Seil Co., Ltd, Shizuoka, Japan, respectively). The urine creatinine concentration was analyzed using the Accuras Auto-Cre diagnosis kit (Shino-Test, Tokyo, Japan) and biopyrrin, 8-OHdG, and cortisol concentrations were corrected based on the urine creatinine concentration. One participant was excluded from the analysis of biopyrrin, 8-OHdG, and cortisol concentrations because her urine was sent to us without having been frozen.

### Questionnaires

A questionnaire about demographic characteristics, the POMS questionnaire (Educational and Industrial Testing Service, San Diego, CA), and the SCL-90-R questionnaire were given to each of the participants. The questionnaire about demographic characteristics included questions about age, race, and education. The POMS questionnaire assesses six mood subscales: tension-anxiety, depression, anger-hostility, vigor, fatigue, and confusion. High vigor scores reflect a good mood or emotion, and low scores on the other subscales reflect a good mood or emotion. Yokoyama et al. previously translated the 65-item scale of the POMS into Japanese and demonstrated the reliability and validity of this Japanese version of the POMS [19]. The Japanese version of the POMS (Kaneko Shobo Co., Tokyo, Japan) was used for the present study. The test taker rates his/her mood over the past seven days on a 5-point scale ranging from “not at all” to “extremely”. The SCL-90-R is a validated and reliable questionnaire that is sensitive to changes in psychological distress. The validity and reliability of the Japanese version of SCL-90-R has been confirmed [20]. It is a 90-item self-report symptom inventory and consists of nine symptom dimensions, somatization, obsessive-compulsive, interpersonal sensitivity, depression, anxiety, hostility, phobic anxiety, paranoid ideation, and psychoticism. The test taker rates how much each of 90 problems had distressed or bothered them in the past seven days on a 5-point scale ranging from “not at all” to “extremely. Somatization, anxiety, depression, and hostility were chosen for study because it has been demonstrated that the practice of yoga improves the mental state by lowering the levels of anxiety, depression and anger-hostility [4,5,21,22] and because we wanted to ensure that the practice of yoga improves somatic symptoms. These questionnaires were chosen because of their sensitivity to change through therapeutic intervention in about 12 weeks [10,22]. Of the participants, the data of three were excluded from the SCL-90-R data analysis because they did not fill out the back page of the questionnaire.

### Statistical analysis and sample size

Statistical analyses were performed using a statistical software package (PASW Statistics 18, version 18.0.0 for Windows; SPSS Inc., Chicago, IL, USA). The Kruskal-Wallis test was used to compare age and education among the yoga-training, control, and long-term yoga groups. Distribution of the subscale scores of POMS and biopyrrin, 8-OHdG, and cortisol concentrations was analyzed using the Kolmogolov-Smirnov test, and we found that the tension-anxiety, depression, anger-hostility, fatigue, and confusion scores of POMS and biopyrrin, 8-OHdG, and cortisol concentrations were not normally distributed. In addition, the anxiety and hostility subscales of SCL-90-R in Japanese community samples had a floor effect (average – 1SD < 0) [20]. Therefore, we chose to run non-parametric statistics. The Wilcoxon signed-rank test was utilized to compare the biopyrrin, 8-OHdG, and cortisol concentrations, which were corrected based on the urine creatinine concentration and the scores of questionnaires before and after 12 weeks of yoga training. The Mann–Whitney U-test was used to compare the biopyrrin, 8-OHdG, and cortisol concentrations and the scores of questionnaires between the control group and the yoga-training group before yoga training and between the long-term yoga group and the yoga-training group after yoga training. Spearman rank correlation was used to test the relationship between the somatization score and the POMS scores or between the changes of subscale scores in POMS and SCL-90-R and the changes of stress-related urinary biomarkers (8-OHdG, biopyrrin, and cortisol). Differences of *p* < 0.05 were considered to be statistically significant.

We estimated that a sample size of 21 would allow us to detect significant differences in the before and after yoga training somatization scores from the SCL-90-R with 80% power (α = 0.05) based on the mean and standard deviation of the somatization scores reported in a previous study of mindfulness [10]. The sample size was calculated using the Power and Sample Size Calculation Software version 3.0 for Windows (Vanderbilt University, Nashville, TN, USA).

## Results

### Follow-up and demographics

The data of 24 of the 39 participants, those who completed at least 10 of the 12 weekly yoga sessions and practiced on their own at home at least twice a week for over 30 minutes during the 12 weeks, were available for analysis (yoga-training group). There were no significant differences in the subscale scores of POMS and SCL-90-R and the stress-related urinary biomarkers before starting yoga training between those who dropped out before the end of the yoga training and those able to complete the 12 weeks of yoga training (data not shown). The demographic data of the yoga-training group, including age, race, and education, are shown in Table 1. As reference data, the demographic data of the long-term yoga group (with more than two years of experience with yoga) and the control group (without experience with yoga) in our previous study [6] are also shown in Table 1. No significant differences were found among the three groups.

**Table 1:** Demographic data of the yoga-training group and the reference data of the control and long-term yoga groups

**Characteristics**	**Yoga-training group**	**Control group**	**Long-term yoga group**
	**(n = 24)**	**(n = 37)**	**(n = 38)**
Age[Mean(SD)]	36.79 (6.43)	34.43 (8.16)	33.84 (7.33)
Range	(25–46)	(22–49)	(22–49)
Ethnicity (%)			
Japanese	100 (n = 24)	100 (n = 37)	100 (n = 38)
Education (%)			
Junior H.S. graduate	0.0 (n = 0)	0.0 (n = 0)	2.6 (n = 1)
H.S. graduate	37.5 (n = 9)	24.3 (n = 9)	23.7 (n = 9)
Junior college graduate	41.7 (n = 10)	37.8 (n = 14)	42.1 (n = 16)
College graduate	20.8 (n = 5)	29.7 (n = 11)	31.6 (n = 12)
Blank	0.0 (n = 0)	8.1 (n = 3)	0.0 (n = 0)

SD: standard deviation. H.S.: high school. The Kruskal-Wallis test was used to compare the age and education levels among the yoga training, control, and long-term yoga groups.

### Psychological distress from the POMS

**Figure 1:**
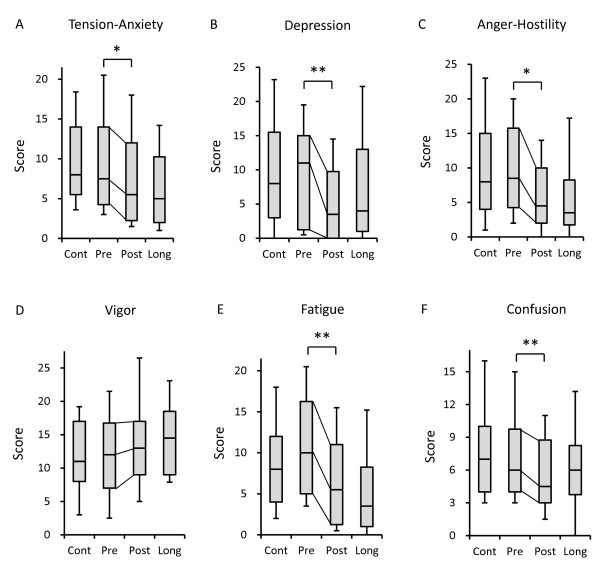
**Psychological distresses results from the Profile of Mood State (POMS) related to yoga training.** Box plot representing the POMS subscale score of the yoga-training group (n = 24) before (Pre) and after (Post) 12 weeks of yoga training with reference control (Cont) and long-term yoga groups (Long). The 25th and 75th percentiles are represented by the lower and upper borders of the grey box. The dark line within the grey box represents the median. The whisker error bars represent the 10th and 90th percentiles. **(A-C, E, F)** All negative subscale scores, tension-anxiety (*p* = 0.022), depression (*p* = 0.010), anger-hostility (*p* = 0.020), fatigue (*p* = 0.001), and confusion score (*p* = 0.004), from the POMS after the 12 weeks of yoga training were significantly decreased compared with those before starting yoga training. **(D)** There was a trend toward an increased vigor (*p* = 0.083). Statistically significant differences are shown: *P < 0.05, **P < 0.01.

The results of the subscale scores of the POMS questionnaire are shown in Figure 1. To add reference for the current results, the subscale scores of the control (without experience with yoga) and long-term yoga (with more than two years of experience with yoga) groups from our previous cross-sectional study are shown on the left and right sides, respectively [6]. All negative subscale scores, tension-anxiety (*p* = 0.022; Figure 1A), depression (*p* = 0.010; Figure 1B), anger-hostility (*p* = 0.020; Figure 1C), fatigue (*p* = 0.001; Figure 1E), and confusion (*p* = 0.004; Figure 1F) of the POMS after the 12 weeks of yoga training were significantly decreased compared with those before starting yoga training. There was a trend toward an increase of the vigor score after the 12 weeks of yoga training (*p* = 0.083; Figure 1D). There were no significant differences in any of the negative subscale scores of the POMS questionnaire between the control group and the yoga-training group before yoga training and between the long-term yoga group and the yoga-training group after yoga training.

### Psychosomatic symptoms from the SCL-90-R and the relationship between the change of somatization and the change of mood state

**Figure 2:**
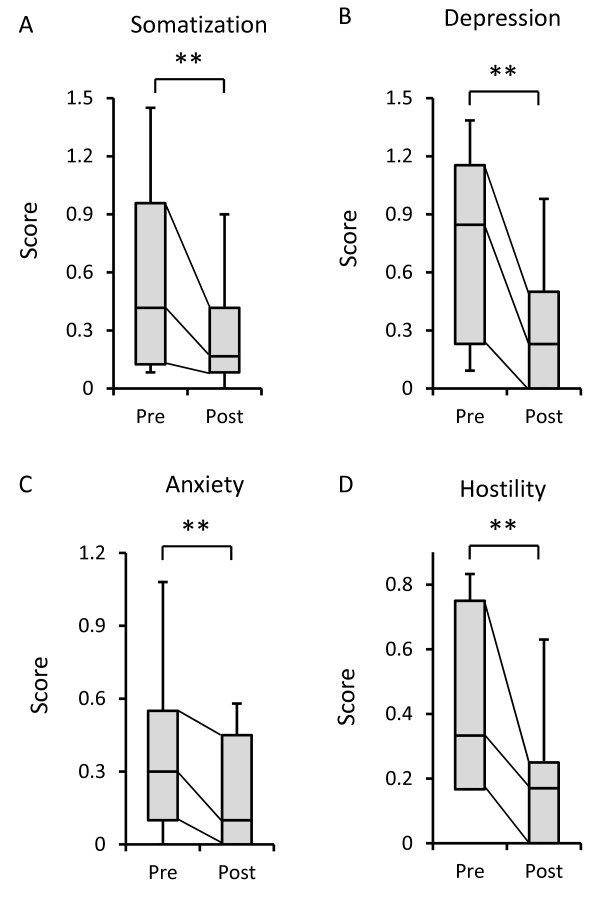
**Somatization and psychological symptoms from the Symptom Check List-90-R (SCL-90-R).** Box plot representing the SCL-90-R subscale scores of the yoga-training group before (Pre) and after (Post) 12 weeks of yoga training (n = 21). The 25th and 75th percentiles are represented by the lower and upper borders of the grey box. The dark line within the grey box represents the median. The whisker error bars represent the 10th and 90th percentiles. **(A-D)** Scores for somatization (*p* = 0.006), depression (*p* = 0.002), anxiety (*p* = 0.002), and hostility (*p* = 0.007) from SCL-90-R were significantly decreased after 12 weeks of yoga training compared with those before starting yoga training. Statistically significant differences are shown: **P < 0.01.

Scores for somatization (*p* = 0.006), depression (*p* = 0.002), anxiety (*p* = 0.002), and hostility (*p* = 0.007) from SCL-90-R were significantly decreased after 12 weeks of yoga training compared with those before starting yoga training (Figure 2A-D).

Therefore, we investigated if changes in mood were related to changes in somatization. However, we did not find any significant correlation between the changes in mood (tension-anxiety, depression, anger-hostility, vigor, fatigue, and confusion in POMS) and the changes in somatization (data not shown). These results suggest that the change of somatization does not have a direct relation with the change of mood.

### Stress-related urinary biomarkers

**Figure 3:**
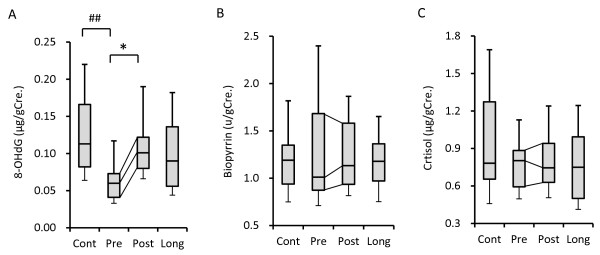
**Change of stress-related urinary biomarkers by 12 weeks of yoga training.** Box plot representing the stress-related urinary biomarkers of the yoga-training group (n = 23) before (Pre) and after (Post) yoga training with reference control (Cont) and long-term yoga groups (Long). The 25th and 75th percentiles are represented by the lower and upper borders of the grey box. The dark line within the grey box represents the median. The whisker error bars represent the 10th and 90th percentiles. **(A)** The urinary 8-Hydroxydeoxyguanosine (8-OHdG) concentration after 12-weeks of yoga training showed a significant increase compared with that before starting yoga training. The urinary 8-OHdG concentration of the yoga-training group before yoga training was significantly lower than that of the control group from our previous study. **(B, C)** No significant changes were observed in the levels of urinary biopyrrin and cortisol after the 12 weeks of yoga training. Statistically significant differences are shown: *P < 0.05 (Wilcoxon signed-rank test), ##*P* < 0.01 (Mann-Whitney U-test).

The stress-related biomarkers urine concentration of 8-OHdG, biopyrrin, and cortisol are shown in Figure 3. As a reference for the current results, the urine concentration of the control group is shown on the left and that of the long-term yoga group is shown on the right [6]. Contrary to our expectation, the urinary 8-OHdG concentration after 12 weeks of yoga training showed a significant increase compared with that before the start of yoga training (Figure 3A). The urinary 8-OHdG concentration of the yoga-training group before yoga training was significantly lower than that of the control group from our previous study (Figure 3A). No significant changes were observed in the levels of urinary biopyrrin and cortisol after the 12 weeks of yoga training (Figure 3B, C). There were no significant differences in the urinary biopyrrin and cortisol levels between the control group and the yoga-training group before yoga training and between the long-term yoga group and the yoga-training group after yoga training.

### Correlations between the changes of the POMS and SCL-90-R subscale scores and the changes of stress-related urinary biomarkers

We investigated if the changes of the subscale scores of the POMS and SCL-90-R are related to the changes of stress-related urinary biomarkers, such as 8-OHdG, biopyrrin, and cortisol; however, we did not find any significant correlations (data not shown).

## Discussion

After 12 weeks of yoga training, all negative subscale scores (tension-anxiety, depression, anger-hostility, fatigue, and confusion) from the POMS and somatization, anxiety, depression, and hostility from the SCL-90-R were significantly decreased compared with those before starting yoga training. This is the first study to demonstrate the effect of yoga training on the somatization symptoms of healthy women who had almost normal somatization scores on the SCL-90-R (Before yoga: 6.86 ± 6.69, After yoga: 3.64 ± 4.04 in this study and in a Japanese community sample: 7.56 ± 5.64 in a previous study [20]; these figures were calculated as the somatization scores in this paper and the previous paper multiplied by 12, because the number of questions about somatization in SCL-90-R are 12). Our findings suggest that regular yoga training reduces not only the level of psychological symptoms, but also somatization symptoms.

Somatization symptoms include medically unexplained physical symptoms such as headache, dizziness, chest pain, lower back pain, and nausea. Some of these physical symptoms have been shown to improve with yoga training. For example, it has been reported that the practice of yoga improved the symptoms of patients with clinically diagnosed migraine [23] and chemotherapy-induced nausea [24]. However, these symptoms are not classified as somatization symptoms because they can be explained medically. Additionally, there is evidence of lower back pain improvement with yoga training [25,26]. However, low back pain is only one of the 12 somatization score items on the SCL-90-R. A few studies have demonstrated that mindfulness training improves somatization symptoms [9,10]. Meditation is a major component of mindfulness. However, the participants of our study practiced a combination of classical postures, breathing exercises, and meditation. Therefore, our findings show that this combination of yoga practices also has the potential to improve somatization symptoms.

Yoga has been reported to be effective with respect to negative psychological symptoms, such as anxiety [3,27-29], anger/hostility [29], and depression [29-31]. Our previous study also suggested that long-term yoga training can improve negative psychological symptoms such as anxiety and anger [6]. Our current results using the POMS and SCL-90-R questionnaires confirm these previous findings that negative psychological symptoms, such as anxiety, anger/hostility, depression, and confusion, are improved by yoga.

As for the relation between mood and somatization, previous study showed that only anxiety, not depression nor anger, had a direct effect on the somatic symptoms of anxiety disorder patients, whereas anxiety and depression, not anger, had a direct effect on the somatic symptoms of somatoform disorder patients [32]. However, in healthy people there were no significant correlations between the changes in mood (tension-anxiety, depression, anger-hostility, vigor, fatigue, and confusion in POMS) and the changes in somatization. Our results suggested that anxiety, depression, and anger are not intimately linked to somatic symptoms in healthy people.

Contrary to our expectation, the level of urinary 8-OHdG after 12 weeks of yoga training showed a significant increase compared with that before starting yoga training. In Figure 3A, the urinary 8-OHdG level of the participants before starting yoga was significantly lower than that of the control group from our previous study [6] (*p* < 0.01; Mann–Whitney U-test). Before being corrected based on the urine creatinine concentration, the average urinary 8-OHdG level of the participants before starting yoga (7.0 ng/mL) was lower than the reference value for women (10.3 ng/mL) described in the document for the ELISA kit we used that appears on the manufacturing company’s website (http://www.jaica.com/guidance_8ohdg/index.html) Japanese. It is unclear why the urinary 8-OHdG level before starting yoga was lower than that of the control group. It is possible that the yoga-training group had an atypical background in terms of their daily activity. A previous study suggested that a heavy burden of work in addition to daily domestic roles increases urinary excretion levels of 8-OHdG [33]. We speculate that the participants in the yoga-training group, in contrast with the others, had sufficient time to attend and continue the 12 weeks of training sessions. Most of them may have been working-women without daily domestic roles. Viewed in this light, through the practice of yoga, their activity levels may have increased and approached the activity levels of the long-term yoga group. Further studies are needed to elucidate the relationship between urinary 8-OHdG and psychosomatic distress that include the activity level.

The analysis of urinary biopyrrin in our previous cross-sectional study of yoga indicated no significant differences in the level of urinary biopyrrin between the control and long-term yoga groups [6]. The current results also showed no significant change in urinary biopyrrin levels after 12 weeks of yoga training. This may be because it is difficult for healthy people to reduce their concentration of urinary biopyrrin by yoga training because their urinary excretion of biopyrrins is already stable at a lower concentration. Our results suggest that biopyrrin is suitable for studies of clinical populations, but not suitable for studies of healthy people.

Cortisol is an accepted, objective stress-related biomarker, because dysregulation of the level of cortisol is related to pathologies associated with stress-related symptoms, such as anxiety, depression, and negative affect [34-36]. However, a previous cross-sectional study of meditation and our previous cross-sectional study of yoga demonstrated that the baseline cortisol level of long-term practitioners was almost the same as that of a control group [6,37]. Our present results also showed no significant change in urinary cortisol level by 12 weeks of yoga training. Although it has been reported that chronic stress causes a high basal cortisol level, the basal cortisol level of most healthy participants may be stable at a lower concentration. Another possibility is that we did not get the accurate peak cortisol level of all participants because we did not take into consideration differences in the participant’s sleep/wake schedules, although urine was collected between 6:00 and 9:00 am. Circadian rhythm should be tracked leading up to the urine collection to more accurately measure the cortisol level.

This study has some limitations. The first is the small sample size. However, we were able to demonstrate significant differences in somatization and psychological scores because there were more participants than the calculated sample size necessary to insure significance. The second limitation is that there is a lack of a comparison group. By using the previous data as a reference, we were able to show that the data of the yoga-training group before yoga training were almost the same as the data of the control group, except for the level of 8-OHdG. Also, the data of the yoga-training group after yoga training were almost the same as the data in the long-term yoga group. These data indicate that the changes of somatization and psychological symptoms in the yoga-training group compared with the “true” control group would have remained significant if an RCT has been used. Despite these limitations, the present findings suggest that yoga training can reduce the somatization score and scores related to mental health indicators, such as anxiety, depression, anger, and fatigue. Yoga training may affect both physical and mental well-being and be useful for preventing somatization and mental disorders. Further studies that measure psychosomatic symptoms and stress-related biomarkers of patients with somatization using yoga intervention compared with controls without yoga intervention, taking into consideration differences in their sleep/wake schedules using methods such as 24 hr urine collection, and/or sleep/wake logs, are needed to verify the effect of yoga.

## Conclusions

Yoga training has the potential to reduce the somatization score and scores related to mental health indicators, such as anxiety, depression, anger, and fatigue. The present findings suggest that yoga can improve somatization and mental health status and has implications for the prevention of psychosomatic symptoms in healthy women.

## Abbreviations

POMS: Profile of mood state; SCL-90-R: Symptom Checklist-90-Revised; 8-OHdG: 8-hydroxydeoxyguanosine; RCT: Randomized control trial; SD: Standard deviation; H.S.: High school.

## Competing interests

The authors declare that they have no competing interests.

Our interpretation of the data and presentation of the information was not influenced by the Japan Yoga Therapy Society, which funded our study.

## Authors’ contributions

K.Y. conceived the study, participated in the design of the study, carried out data collection, performed the statistical analysis and drafted the manuscript. T.H. participated in the design of the study and carried out data collection. T.O. and N.S. evaluated the results of the study and reviewed the manuscript. C.K. participated in the design and coordination of the study and reviewed the manuscript. All authors read and approved the final manuscript.
